# User involvement in adolescents’ mental healthcare: a systematic review

**DOI:** 10.1007/s00787-021-01818-2

**Published:** 2021-06-05

**Authors:** Petter Viksveen, Stig Erlend Bjønness, Nicole Elizabeth Cardenas, Julia Rose Game, Siv Hilde Berg, Anita Salamonsen, Marianne Storm, Karina Aase

**Affiliations:** 1 SHARE – Centre for Resilience in Healthcare, Faculty of Health Sciences, University of Stavanger, 4036 Stavanger, Norway; 2 Department of Quality and Health Technology, Faculty of Health Sciences, University of Stavanger, 4036 Stavanger, Norway; 3 Department of Psychiatry, Stavanger University Hospital, Stavanger, Norway; 4 Faculty of Health Sciences, University of Aberdeen, Aberdeen, Scotland; 5 Faculty of Medicine, Pomeranian Medical University in Szczecin, Szczecin, Poland; 6 Regional Centre for Child and Youth Mental Health and Child Welfare - North (RKBU North), Faculty of Health Sciences, UiT The Arctic University of Norway, Langnes, P.O. Box 6050, Tromsø, Norway; 7 Department of Public Health, Faculty of Health Sciences, University of Stavanger, Stavanger, Norway

**Keywords:** User involvement, Adolescents, Mental healthcare, Systematic review

## Abstract

More than one out of ten adolescents suffer from mental illness at any given time. Still, there is limited knowledge about their involvement in mental healthcare. Adolescents have the right to be involved in decisions affecting their healthcare, but limited research focuses on their engagement and decision-making. Therefore, this systematic review aims to explore the existing experiences with, the effectiveness of, and safety issues associated with user involvement for adolescents’ mental healthcare at the individual and organizational level. A systematic literature review on user involvement in adolescents’ mental healthcare was carried out. A protocol pre-determined the eligibility criteria and search strategies, and established guidelines were used for data extraction, critical appraisal, and reporting of results. Quantitative studies were analysed individually due to heterogeneity of the studies, while qualitative studies were analysed using thematic synthesis. A total of 31 studies were included in the review. The experiences with user involvement were reported in 24 studies with three themes at the individual level: unilateral clinician control versus collaborative relationship, capacity and support for active involvement, the right to be involved; and two themes at the organizational level: involvement outcomes relevant to adolescents’ needs, conditions for optimal involvement. The effectiveness of user involvement was reported in seven studies documenting fragmented evidence related to different support structures to facilitate adolescents’ involvement. The safety associated with user involvement was not reported in any studies, yet a few examples related to potential risks associated with involvement of adolescents in decision-making and as consultants were mentioned.

**Supplementary Information:**

The online version contains supplementary material available at 10.1007/s00787-021-01818-2.

## Introduction

Mental health disorders among adolescents represent long-lasting consequences at an individual level and significant economic and public health challenges. They are associated with poorer physical, sexual and social health; limited social networks; poorer education; and lower employment rates [1–4]. Mortality and suicide rates are higher among those who have mental health disorders compared to other adolescents [5, 6]. Many mental disorders in adults have their onset in childhood or adolescent years [2, 7]. More than one out of ten adolescents suffer from mental illness at any given time, but only a minority seek help and many of those who are offered treatment drop-out [4, 6]. Adolescents have the right to access high quality and safe healthcare services [8, 9]. Moreover, they have the right to be actively involved in their treatment. This implies that they should be heard, their preferences should be considered, and they should take part in decision-making processes affecting their health [10]. National governments committed themselves to strengthen adolescents’ right to be heard in matters affecting their life and health and to participate in decision-making processes, as laid out in the United Nations (UN) General Assembly’s Special Session on Children in 2002. User involvement can take place at the individual level, for adolescents to be involved in activities to plan, deliver or review mental health services for their own healthcare; or at the organizational or systems-level for planning, delivering or reviewing healthcare services for other adolescents’ mental health, including to develop new or to improve existing services; or at the political level to influence policy decisions, e.g. to develop regulation [11–13].

There is limited knowledge about the existing research in the field of user involvement in adolescents’ mental healthcare. A literature review published by in 2005 found that adolescents wanted to be involved in decisions affecting their healthcare [14]. However, at that time involvement of adolescents in their mental healthcare was not so common, and there was limited research assessing it, both in the individual adolescents’ mental healthcare and in service development. User involvement has become more prevalent in mental healthcare. Nevertheless, a systematic review carried out in 2012 found only a handful of studies focusing on adolescents’ engagement and decision-making in healthcare, and none of those focused on mental healthcare [15]. A scoping review identified some approaches to promote shared decision-making in child mental health, including therapeutic techniques; psychoeducation; discussion prompts; aids for planning, setting goals and making decision; and mobilizing patients to engage. However, evidence of the effectiveness of these approaches was limited and it did not assess the wider context of user involvement, beyond decision-making at the individual level [16]. Furthermore, Liverpool et al. [17] identified decision support interventions for parents of children with attention deficit hyperactivity disorder (ADHD), autistic spectrum disorder (ASD), emotional and behavioural problems including depression (EBD), self-harm or universal mental healthcare. Face-to-face, digital and paper-based interventions were found, e.g. to present treatment options, discuss pros and cons, explore values and preferences, and make recommendations. However, the focus of this review was on interventions for parents, rather than for adolescents themselves. Furthermore, clinicians may also be reluctant to change their practice to introduce shared decision-making [18]. No systematic review has explored user involvement in adolescents’ mental healthcare focusing specifically on adolescents’ own involvement in their care and for improving mental health services.

This systematic review fills this knowledge gap with the aim to explore existing experiences with, the effectiveness of, and safety issues associated with user involvement for adolescents’ mental healthcare, at the individual and organizational level [19]. By experiences, we mean adolescents’, healthcare personnel’s or other stakeholders’ descriptions of involvement of adolescents in planning, delivery or review of mental health services for adolescents’ own healthcare (individual level), or for planning, delivering or reviewing mental health services for other adolescents (organizational level). Such experiences could be gathered using qualitative research methods, for example through individual or group interviews. By effectiveness of user involvement, we mean the effect of involvement of adolescents, either at the individual or at the organizational level, measured on specific outcomes assessing involvement itself or health outcomes. Effectiveness could be assessed using controlled or uncontrolled quantitative research designs, e.g. randomized controlled trials, non-randomized controlled trials or uncontrolled studies using quantitative outcome measures. Safety of user involvement could include either adolescents’ or other stakeholders’ descriptions of experiences (in qualitative studies) or outcome measures (in quantitative studies) suggesting negative impact on adolescents’ mental health or safety issues potentially affecting other adolescents or the services themselves such as breach of confidentiality or other violations of General Data Protection Regulations.

This systematic review will contribute to inform clinical practice to determine acceptable, effective, and safe ways of involving adolescents in their healthcare, as well as for developing and improving mental health services.

## Methods

The protocol for this systematic review pre-determined the eligibility criteria, search strategies, guidelines for data extraction, critical appraisal, data synthesis and reporting of results [19].

## Inclusion criteria

We included research articles reporting on involvement of adolescents in mental healthcare at the individual and/or organizational level. Included publications had to fulfil all the criteria presented in Table 1. We used a broad definition of *“user involvement”* as there is no consensus on how the term should be understood and we attempted to include all articles that could contribute to expand current knowledge in this underexplored field of research. Involvement of adolescents could include gathering their experiences, views and perspectives as part of planning, delivering or reviewing their own (individual level) or other adolescents’ (systems-level) mental healthcare. Communication alone, e.g. between adolescents and health personnel during a therapy session, was not sufficient to be considered “*user involvement*”. Health personnel were understood as any person working as an individual practitioner or employees of a health institution (e.g. MeSH Unique ID D006282). Only original articles were included. Literature reviews were only used to identify additional relevant research articles. The inclusion of Nordic languages was due to the rising focus on user involvement in mental healthcare in the Nordic countries over the past decades, which also is the context within which we carry out our research. The period (2002–2019) was set to cover the literature most relevant to current clinical practice and following the 2002 UN General Assembly’s Children’s rights policy.

**Table 1:** Article inclusion criteria

Inclusion category	Category description	Notes
Adolescents	Majority within age range 13–18 years (MeSH Unique ID: D000293)	Included if more than 50% of the participants were within the age range
Study participants	Any participants reporting on adolescents’ involvement in mental healthcare	E.g. adolescents, caretakers, healthcare professionals
Mental healthcare	Healthcare services providing preventive or therapeutic interventions for diagnosed or self-reported mental health and/or substance use problems	Based on MeSH Unique ID: D003191
User involvement (individual level)	Involvement of the individual adolescent in her or his own mental healthcare	Experiences, views and wishes to plan, deliver, review or make other decisions affecting adolescents’ mental healthcare
User involvement (organizational level)	Adolescents’ experiences, views and wishes used to plan, deliver or review mental health services for adolescents in general, including to develop new or to improve existing services	Including adolescents’ experiences with mental health services used in practice implementation or testing in research
Research methods	Studies using qualitative, quantitative or mixed methods	
Publication types	Peer-reviewed and grey literature	Grey literature: academic theses and dissertations; conference abstracts, proceedings, papers; governmental and non-governmental reports
Languages	English, German, French, Danish, Norwegian, Swedish	
Publication year	2002– 2019	

## Search strategy

The literature search strategy included 11 databases and other sources to identify both peer-reviewed articles and grey literature (Table 2). A broad range of search terms were used in order to identify potentially relevant articles reporting on *“user involvement”* as this could include a variety of different activities (Table 2). We customized searches to each database with an aim to maximize sensitivity and specificity. A university librarian was consulted to plan the literature search strategy. Searches were carried out until 16.06.2019 independently by two researchers (PV, SEB), and results were compared. There were minor differences in search results due to searches being carried out a few days apart. Any discrepancies in search results were discussed and all articles identified by at least one of the researchers were included. An example of a full electronic search is presented in Appendix 1. Two researchers (PV, SEB) screened titles and abstracts. The full texts of potentially relevant articles were screened by at least two researchers (AS, PV, KAA, MS, SEB, SHB). Where there were discrepancies in researchers’ assessment, a third researcher and a co-researcher (JRG, NEC) were involved, and consensus for inclusion/exclusion was reached for all articles. All researchers were involved in the full-text screening process (PV, SEB, AS, KAA, MS, SHB). Endnote (version X9) was used to manage data records.

**Table 2:** Literature search strategy

Databases	Academic Search Premier, British Nursing Index, CINAHL, EMBASE, MEDLINE, PsycINFO, PubMed, Scopus, SocINDEX, SveMed + , Web of Science
Other sources	Google Scholar: 50 first results for each search string Researchers: authors of included articles were contacted Mental health organizations Hand search of reference lists of reviews and included articles
Search terms 1: Subject and MeSH terms	User group and field of health: adolescent psychiatry; adolescent psychology Field of research: clinical decision-making; community participation; consumer participation; cooperative behaviour; decision-making; decision-making, organizational; information dissemination; information sharing; patient participation; personal autonomy, public opinion; self-determination
Search terms 2: title search terms	User group: adolescents; teenagers; youth Field of health: mental; psychology; psychiatry
	Field of research: autonomy; client-centred; collaboration; consultation; contribution; decision-making; empowerment; engagement; governance; inclusion; information sharing; involvement; mutual agreement; negotiation; opinions; participation; partnership; patient-centred; peer support; perspectives; self-determination

## Data extraction

Data extractions guidelines included the Critical Appraisal Skills Programme (CASP) for qualitative studies [20], the Cochrane Consumers and Communication Review Group’s data extraction template for trials [21], and the STROBE statement checklist for cohort, case–control and cross-sectional studies [22]. Included articles were divided between the six researchers for data extraction (PV, SEB, AS, KAA, MS, SHB). Data was extracted by one researcher and checked by a second researcher. Agreement on data extraction was reached between researchers. Where available, main outcomes were reported for studies using quantitative methods. For articles reporting multiple outcomes, only those of relevance to the systematic review were included. Information on what data were reported is provided under the section entitled "Reporting of results".

## Quality appraisal

Studies using quantitative methods were assessed using the Cochrane Collaboration’s guidelines for assessment of risk of bias [23]. This included assessment of risk of selection, performance, detection, attrition and reporting bias (each assessed as either low, high or unclear risk of bias); as well as the potential influence of confounding factors for non-randomized studies, as suggested by Reeves et al. [24]. The risk of meta-bias (publication bias across studies and selective reporting within studies) [25] was considered by searching for unpublished studies in the grey literature, and through comparison of the methods and the results sections of included studies when no protocol articles were found. The applicability and generalizability of results of quantitative studies was considered using the Pragmatic Explanatory Continuum Indicator Summary (PRECIS) tool [26]. 

Studies using qualitative methods were appraised using the Critical Appraisal Skills Programme (CASP) to determine rigour, credibility and relevance of the research [20]. Each CASP item was assessed and considered satisfactory (“yes”), not satisfactory (“no”), or providing insufficient information to be assessed (“unclear”). Study quality categories were scored as suggested by others [27], depending on the number of items scoring “yes” (low: 0–5, moderate: 6–8, high: 9–10 items).

## Reporting of results

Results of the literature search are presented using the Preferred Reporting Items for Systematic reviews and Meta-Analyses (PRISMA) flow diagram [28]. Data from randomized controlled trials are reported using the Consolidated Standards of Reporting Trials (CONSORT) statement [29], the Strengthening the Reporting of Observational Studies in Epidemiology (STROBE) statement for observational (cohort and cross-sectional) studies [22], and the CASP checklist for qualitative studies [20].

Characteristics of qualitative studies were tabulated to provide information about participant characteristics (age, gender, mental health status/conditions/problems) with number of participants; intervention/treatment and study setting, methods (research design, recruitment methods, data collection, analytic method); the level of involvement (individual, organizational); and the overall result of a quality assessment. We also report the result of a thematic synthesis of qualitative studies.

Characteristics and results of quantitative studies were tabulated to include information on study design; participant characteristics; interventions and study setting; trial arms, with number of participants in each arm; results of studies, with focus on outcomes of relevance to the systematic review; and assessment of internal validity focusing on risk of bias [23] and external validity, using the PRECIS tool for assessing studies on a pragmatic-explanatory continuum [26]. Key characteristics and results of quantitative studies are also presented for each individual study in text, but with no synthesis of data due to the heterogeneity of the identified studies.

## Thematic synthesis of qualitative studies

A thematic synthesis was developed to report on experiences with involvement of adolescents at the individual and the organizational level. The purpose of the thematic synthesis was to analyse results across different contexts and participants, and to go beyond the content of the original studies to possibly develop new explanations, constructs or hypotheses [30, 31]. The analysis draws on techniques used in thematic analysis, and suggests that the results may be more than merely the sum of the individual studies [32]. Three researchers (KAA, PV, SEB) carried out the thematic synthesis process, but consulted with the co-researchers (JRG, NEC) who contributed to revising the themes. We used the approach suggested by Thomas and Harden [31], by initially identifying all the text in the “results” or “findings” sections of abstracts and full texts of the included qualitative studies. The selected text was marked line-by-line by one researcher (PV) to identify codes of potential relevance to the research aim, and checked by a second researcher (SEB). Coding was discussed and consensus was reached. This was followed by development of descriptive themes, based on codes and developed through an inductive process using no prior theoretical model, but by using the definition described in the inclusion criteria.

The analytic process included analysis and re-analysis through several phases where we revisited the original studies, reassessed the extracted data, and reconsidered codes, themes and descriptions of themes. This could include abandoning preliminary themes or sub-themes that only to a limited extent were supported by extracted data. For example, "leadership support" served as one of the preliminary themes for user involvement at the organizational level but was in the final analysis included in the theme of "conditions for optimal involvement".

## Results

The results were divided into three main sections: (1) experiences with user involvement, reported through studies using qualitative research methods; (2) effectiveness of user involvement, reported through studies using quantitative research methods; and (3) safety associated with user involvement, reported in either qualitative or quantitative studies. First, search results, sources, characteristics, and quality assessment of the included articles are reported.

### Literature search results

**Fig. 1:**
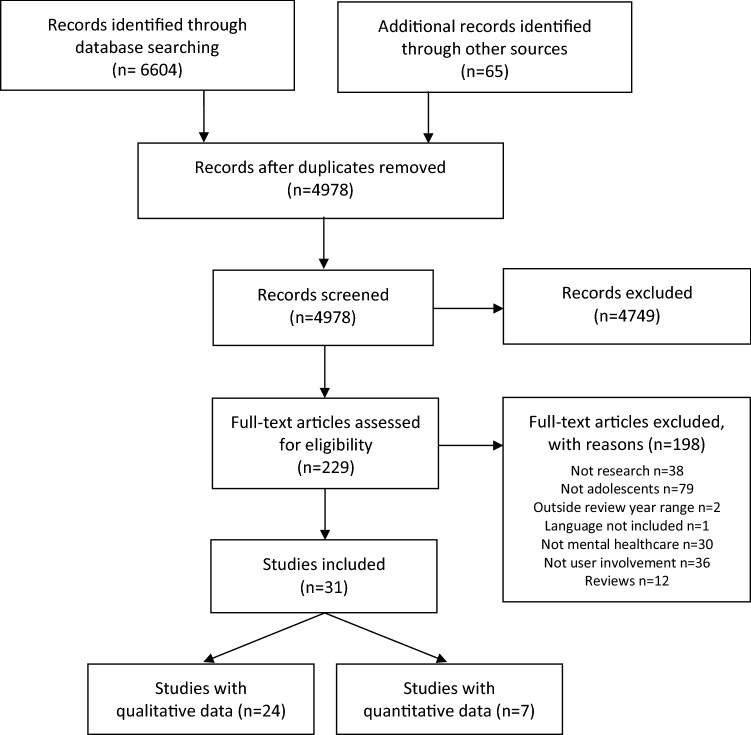
Systematic review PRISMA flow diagram

A total of 4,978 titles were identified through 11 databases and other sources, and 31 articles were included in the systematic review. Most articles were excluded during the screening of titles and abstracts, leaving 229 articles for full-text assessment. Consensus on inclusion/exclusion was reached for all except two articles, where a majority vote was used to make a final decision. Adolescent co-researchers were consulted for six articles where there were initial discrepancies in researchers’ assessments. Further details with reasons for inclusion and exclusion of articles are provided in the PRISMA flow diagram (Fig. 1).

### Sources of included articles

Most included articles (*n* = 26) were identified through database searches, but six were only found using other sources (Table 3). No single database identified more than 12 included articles, and half of the included titles were only found through a single source. Four articles were suggested by some of the 22 researchers in the field of user involvement in adolescents’ mental healthcare we contacted, and two titles that were found by searching reference lists of included articles.

**Table 3:** Sources of included articles

Sources	Number of articles	Unique source^a^
**Tota﻿l**	**31**	**15**
Databases	26	9
PsycINFO	12	2
EMBASE	10	3
Academic Search Premier	9	0
CINAHL	9	0
Web of Science	9	0
MEDLINE	5	1
PubMed	5	1
British Nursing Index	3	1
SocINDEX	3	0
Scopus	1	1
SveMed +	0	0
**Other**	**7**	**6**
Researchers	5	4
Reference lists	2	2
Google Scholar	0	0
Mental health organizations	0	0

^a^Number of articles only identified through a single source

### Characteristics of qualitative studies

Twenty-four studies reporting on qualitative data were included, with a total of 587 participants (median 22, IQR 15–30) (Table 4). The majority of participants were adolescents (*n* = 491, 84%), whereas the remaining were parents, guardians or care providers (*n* = 64, 11%), and healthcare staff (*n* = 32, 6%), reporting on adolescent involvement. Although there was considerable variation in adolescents’ gender distribution between studies (female range 20%–100%), the overall proportion of females and males was equal. Studies were carried out within a wide range of primary and secondary healthcare services (details in Table 4). Most studies (*n* = 15) included either adolescents with specified diagnosed mental health conditions, such as depression, eating disorders, and ADHD; or adolescents with self-reported mental health problems including self-harm, suicidal thoughts or behaviours, and drug or alcohol problems. Mental health problems were not specified in the remaining nine studies. User involvement at the individual level was reported in 17 studies and at the organizational level in 11 studies (four at both levels).

**Table 4:** Characteristics of qualitative studies

Reference	Participant characteristics^a^	Intervention/treatment, study setting	Methods^b^	Involvement level	Quality assessment (CASP)
Bjønness 2015 [54], Norway	*n* = 14, age ≥ 16 years (girls *x̄* = 18.2, boys *x̄* = 17.3), female 64%, mental health conditions (unspecified)	Treatment unspecified, child and adolescent outpatient mental health services, specialist care	Qualitative study Convenience sample, recruited by therapists Semi-structured interviews Systematic text condensation	Individual	High
Block 2013 [57], USA	*n* = 25, age 12–17 years, female 44%, mental health conditions (unspecified)	Treatment unspecified, outpatient mental health services	Qualitative study Consecutive sample referred from schools to mental health services, participation rate: 32% (25 of 78) Semi-structured interviews Grounded theory analysis	Individual	Moderate
Boydell 2010 [40], Canada	*n* = 30, age 13–18 years (*n* = 19), 7–12 years (*n* = 11), female 43%, conditions: ODD, ADHD, mood disorder, learning disability, anxiety disorder, conduct disorder, attachment disorder, developmental disability, foetal alcohol effects, adjustment disorder	Psychiatric consultations using interactive video conferencing technology, University division of child psychiatry with training sites at children’s, teaching and community hospitals	Qualitative study Recruitment strategy not specified Individual interviews Interpretive interactionist framework analysis	Individual & Organizational	Moderate
Coates 2014 [55], Australia	*n* = 12, age 15–23 years (*x̄* = 18.9), female 58%, conditions: anxiety, depression, PTSD, eating disorder, borderline personality disorder	Treatment unspecified, offered by foundation providing services for youth with mental health and/or drug and alcohol issues, governed under services provided by the local health district, primary care	Qualitative study Recruitment of new youth alliance members joining a national youth mental health foundation through advertising and information sessions Focus group interview Analytic approach not described	Organisational	Moderate
Coates 2016 [59],^**c**^ Australia	*n* = 15, adolescents *n* = 12, managers *n* = 3, adolescents: age 15–23 years (*x̄* = 18.9), female 58%, conditions: anxiety, depression, PTSD, eating disorder, borderline personality disorder	Treatment unspecified, offered by foundation providing services for youth with mental health and/or drug and alcohol issues, governed under services provided by the local health district (primary and secondary care)	Qualitative study Recruitment of new youth alliance members joining a national youth mental health foundation through advertising and information sessions Focus group interviews: adolescents (*n* = 3), management (*n* = 1), supplemented with documents including model descriptions and youth activity logs Thematic analysis	Organizational	Moderate
Coyne 2015 [41], Ireland	*n* = 47, adolescents *n* = 15, parents: *n* = 32, adolescents: age 11–17 years, female 60%, conditions: mood disorder, ADHD, impulse control, anxiety, adjustment and behavioural disorders	Treatment unspecified, provided in three Child and Adolescent Mental Health Services (CAMHS) clinics	Qualitative study Recruitment by a clinician within the service Individual and focus group interviews Thematic analysis	Individual	High
Crickard 2010 [50], USA	*N* = 17, adolescents *n* = 6, parents/guardians *n* = 6, staff *n* = 5, adolescents: age 14–17 years, gender not specified, mental health conditions (unspecified)	Treatment unspecified, community mental health centre	Qualitative study Recruitment not described Individual interviews Analytic method not reported	Individual Organizational	Low
Forchuk 2016 [63], Canada	*N* = 46, adolescents *n* = 37, care providers *n* = 9, adolescents: age 16–21 years (*x̄* = 17, SD 1.4), female 73%, conditions: symptoms of depression, comorbidities: anxiety disorder, mood disorder, eating disorder, psychotic disorder, personality disorder	Web-based application that allows adolescents to create and manage an electronic personal health record	Mixed methods, but only qualitative data used for the systematic review Recruitment through care providers working in acute and tertiary care facilities Focus group interviews Thematic analysis according to Leininger’s phases of qualitative data analysis	Organizational	Moderate
Graham 2014 [51], UK	*N* = 50, age 16–25 years (16-17y *n* = 22, 16-19y *n* = 6), within in review age range: female 54%, unspecified self-reported mental health problems in 46% (*n* = 13)	Treatment unspecified, various service use, primary care	Mixed methods, but only qualitative data used for the systematic review Snowballing recruitment through two GP practices, three CAMHS, student counselling service, homeless shelter, supported housing project Focus group and individual interviews, participatory research groups, nominal group technique Thematic analysis and nominal group technique	Individual	High
Gros 2017 [53], Canada	*N* = 6, age 13–18 years, female 67%, conditions: psychosis, mood disorders, borderline personality disorders, eating disorders, suicide risk	Treatment unspecified (min. 3 days) in acute inpatient psychiatric unit and in a day unit	Qualitative study Convenience sampling Semi-structured interviews and observations of participants’ non-verbal behaviour and contextual information Constant comparative analysis method	Individual Organizational	High
Hart 2005 [48], UK	*N* = 27, age 11–18 years, female 59%, conditions: depression, school behavioural difficulties, ADHD, self-harm, family breakdown	Treatment unspecified, child and adolescent mental health services (range: < 1 year to 8 years), primary care	Qualitative study Recruited by therapists in specialist CAMHS Home interviews with adolescents & their parents, and focus group interviews (girls, boys & parents separately) Analysis method unclear, possibly thematic	Individual	Moderate
Latif 2017 [60], UK	*N* = 11, adolescents: *n* = 4, nurses *n* = 7, adolescents: age 10–18 years (*x̄* = 15), female 100%, self-harm injuries	Treatment unspecified, inpatient acute care services/hospital	Qualitative study Recruitment from CAMHS Workshops with story boards Delphi technique	Organizational	Moderate
LeFrancois 2007 [42], UK	*N* not specified, age 11–18 years, gender not specified, mental health conditions unspecified	Treatment unspecified, adolescent psychiatric inpatient unit/hospital	Qualitative study Recruitment method unclear Semi-structured and unstructured individual and group interviews, adolescents’ self-recorded unstructured conversations, additional written material (e.g. personal diaries, poetry, cards, drawings), over 4 months Ethnographic study, analysis method unclear	Individual	Low
LeFrancois 2008 [43], UK	*N* not specified, age 11–18 years, gender not specified, mental health conditions unspecified	Treatment unspecified, adolescent psychiatric inpatient unit/hospital	Qualitative study Recruitment method unclear Semi-structured and unstructured individual and group interviews, observation of conversations and interactions between practitioners and adolescents, investigation of written material (e.g. patient files, diaries, internal policy documents), over 4 months Ethnographic study, analysis method unclear	Individual	Moderate
Manning 2016 [46], UK	*N* = 8, age 10–18 years, gender not specified, conditions: self-harm, eating disorders	Treatment unspecified, acute inpatient care for adolescents with mental health problems, psychiatric unit/hospital	Qualitative study Recruitment from a tertiary children’s hospital, recruitment rate 13% (8 out of 63 invited) Nominal group technique: Participant generated statements related to their experiences Thematic analysis	Individual	Moderate
Moses 2011 [47], USA	*N* = 80, age 13–18 years (*x̄* = 15.6), female 61%, hospitalization reasons: suicidal ideation or non-suicidal self-harm (63%), suicide attempts (19%), aggression or out-of-control behaviour incl. substance use (13%), medication assessment or school refusal (6%)	Treatment unspecified, psychiatric inpatient treatment/hospital	Qualitative study Recruitment through hospital admission staff Semi-structured individual interviews Thematic analysis with constant comparative method	Individual	High
Nadeau 2017 [56], Canada	*N* = 15, adolescents *n* = 5, parents *n* = 5, clinicians *n* = 5, adolescents: age 12–17 years (*x̄* = 13.6,SD2.0), female 20%, conditions: emotional external behaviour problems, depression, ADHD	Treatment unspecified, free local community health centres (CLSC)	Qualitative study Recruitment through primary care clinicians Semi-structured individual interviews Thematic analysis	Organizational	Moderate
Oruche 2014 [44], USA	*N* = 24, adolescents *n* = 12, caregivers *n* = 12, adolescents: age 13–17 years, gender not specified, mental health treatment, conditions unspecified	Treatment unspecified, community mental health centre, primary care	Qualitative study Recruitment through community mental health centre, recruitment rate: 60% (12 of 20) Focus group interviews with adolescents (*n* = 2) and caregivers (*n* = 2) (separately) Content analysis	Individual	Moderate
Ranney 2015 [58], USA	*N* = 21, age *x̄* = 15.3 years, female 57%, depression symptoms (PHQ-9 *x̄* = 11.3,SD6.5) and peer violence (CTS-2 *x̄* = 11.0,SD9.5)	Text-message-based depression prevention intervention, primary & secondary care	Qualitative study Recruitment of consecutive adolescents at trauma paediatric emergency department, children’s hospital Semi-structured individual interviews Thematic analysis	Individual	High
Rodarmel 2014 [45], USA	*N* = 30, age 14–21 years, gender not specified, mental health conditions unspecified	Treatment unspecified, school-based mental health services, primary care	Qualitative study Recruitment through school-based hospitalization services (*n* = 26), and youth involvement and family–school–community partnership groups (*n* = 4) Open-ended narrative surveys Grounded theory study	Individual Organizational	Moderate
Stockburger 2005 [61], Canada	*N* = 21, age 15–19 years, gender not specified, experiences with drugs and alcohol	Treatment unspecified, local drug and alcohol treatment and support programmes	Qualitative study Recruitment through local youth-serving agencies Focus group interviews (*n* = 4) Thematic analysis	Organizational	Moderate
Sundar 2012 [52], Canada	*N* = 25, adolescents *n* = 13, practitioners *n* = 12, adolescents: age 16–20 years, female 62%, mental health conditions unspecified, use or have used mental health services	Treatment unspecified, mental health services, primary & secondary care	Qualitative study Recruitment of convenience sample, recruitment method not reported Focus group interviews with youth (*n* = 2) and practitioners (*n* = 2) Grounded theory approach, constant comparison method	Individual	High
Thorsen 2018 [62], USA	*N* = 41, age 13–17 years; group A: *n* = 20, age *x̄* = 15.4,SD1.4, female 100%, group B: *n* = 21, age *x̄* = 15.3,SD1.2, female 43%; at risk of depression and victim or perpetrator of physical peer violence	Preventive CBT-based SMS-delivered intervention, emergency department in children’s hospital	Qualitative study Recruitment from an urban emergency department Semi-structured interview Thematic analysis	Organizational	Moderate
Wisdom 2006 [49], USA	*N* = 22, individual interviews *n* = 15: age 14–19 years (*x̄* = 16.3), female 53%, focus group participants *n* = 7: age 15 years, female 71%, diagnosis: major depression, dysthymia or depression not otherwise specified	Current or past psychotherapy and/or antidepressants, or no treatment, primary care	Qualitative study Recruitment: individual interviews through primary care practitioner, focus group interview: through a high school Individual (*n* = 15) and focus group interviews (*n* = 1) Grounded theory approach, constant comparison method	Individual	High

^a^Participant characteristics includes age, gender, mental health status/conditions/problems

^b^Methods include research design, recruitment methods (for adolescents), data collection and analytic method. The reported design refers to the approach used to collect data of relevance to the review

^c^The first focus group interview included in Coates 2016[59] was also reported on in Coates 2014[55]

### Quality assessment of qualitative studies

All studies satisfied the first two criteria of the Critical Appraisal Skills Programme (CASP) guidelines [20], including a clear aim of the research and the appropriateness of using qualitative methodology to address the research goal (Table 5). The CASP guidelines suggest that it is then worth proceeding with an assessment of the remaining questions.

**Table 5:** Quality assessment of qualitative studies

Main author, year	1	2	3	4	5	6	7	8	9	10	Involvement level ^a^	Assessment (CASP) ^b^
Bjønness 2015 [54]	Y	Y	Y	Y	Y	Y	Y	Y	Y	Y	I	High
Block 2013 [57]	Y	Y	Y	Y	Y	U	Y	U	Y	U	I	Moderate
Boydell 2010 [40]	Y	Y	Y	U	Y	U	N	Y	Y	Y	I/O	Moderate
Coates 2014 [55]	Y	Y	U	Y	Y	U	Y	U	Y	U	O	Moderate
Coates 2016 [59]	Y	Y	Y	Y	Y	N	Y	U	Y	Y	O	Moderate
Coyne 2015 [41]	Y	Y	Y	Y	Y	N	Y	Y	Y	Y	I	High
Crickard 2010 [50]	Y	Y	Y	U	Y	U	N	U	N	Y	I/O	Low
Forchuk 2016 [63]	Y	Y	Y	U	Y	U	U	U	Y	Y	O	Moderate
Graham 2014 [51]	Y	Y	Y	Y	Y	Y	Y	Y	Y	Y	I	High
Gros 2017 [53]	Y	Y	Y	Y	Y	Y	Y	Y	Y	Y	I/O	High
Hart 2005 [48]	Y	Y	Y	Y	Y	N	Y	U	Y	Y	I	Moderate
Latif 2017 [60]	Y	Y	Y	U	Y	U	Y	U	Y	Y	O	Moderate
LeFrancois 2007 [42]	Y	Y	Y	U	Y	U	N	U	Y	U	I	Low
LeFrancois 2008 [43]	Y	Y	Y	U	Y	U	N	U	Y	Y	I	Moderate
Manning 2016 [46]	Y	Y	Y	Y	Y	N	N	U	Y	U	I	Moderate
Moses 2011 [47]	Y	Y	Y	Y	Y	N	Y	Y	Y	Y	I	High
Nadeau 2017 [56]	Y	Y	Y	Y	Y	U	Y	Y	Y	U	O	Moderate
Oruche 2014 [44]	Y	Y	Y	Y	Y	U	Y	Y	Y	U	I	Moderate
Ranney 2015 [58]	Y	Y	Y	Y	Y	N	Y	Y	Y	Y	I	High
Rodarmel 2014 [45]	Y	Y	Y	Y	Y	NA	Y	Y	Y	U	I/O	Moderate
Stockburger 2005 [61]	Y	Y	Y	U	Y	Y	Y	U	Y	Y	O	Moderate
Sundar 2012 [52]	Y	Y	Y	Y	Y	N	Y	Y	Y	Y	I	High
Thorsen 2018 [62]	Y	Y	Y	Y	Y	U	N	Y	Y	Y	O	Moderate
Wisdom 2006 [49]	Y	Y	Y	Y	Y	U	Y	Y	Y	Y	I	High

a. I = Individual level, 0 = Organizational level. b. CASP criteria are presented in appendix 2. Y = Yes, N = No, U = Unclear, NA = Not applicable. Scoring: Low: Studies meeting 0–5 of the CASP

checklist criteria. Moderate: studies meeting 6–8 of the criteria. High: studies meeting 9–10 of the criteria. For question 10, the score was considered to be Yes if the study was considered to be of "relevance" or "some relevance" to the systematic review, and Unclear if it was considered to be of "limited relevance"

Overall, most studies (*n* = 14) were of moderate quality, one-third were of high quality, and two studies were of low quality. The most common weakness in the studies was a lack of consideration or reporting of the relationship between the researchers and the participants, which was only adequately done and sufficiently described in four studies. Other prevalent limitations included a lack of rigour in reporting of data analysis methods (*n* = 8), participant recruitment strategies (*n* = 7), and consideration of ethical issues (*n* = 7). A complete overview of CASP questions and criteria may be found in Appendix 2.

### Characteristics of quantitative studies

Seven studies used quantitative methods, out of which six reported on user involvement at the individual level [33–38], and one at the organizational level [39]. This included a single randomized controlled trial [38]; a non-randomized comparative study [36]; two longitudinal prospective cohort studies [33, 34]; a cohort study using pre- to post-assessment [37]; and two cross-sectional surveys [35, 39], out of which one also used repeated measures for some participants [39]. There was considerable heterogeneity between studies. Further study characteristics are presented in Table 6.

**Table 6:** Effectiveness of user involvement in adolescent mental healthcare

Reference	Study design	Participant characteristics	Intervention, study setting	Trial/study arms^a^	Results	Internal and external validity assessment ^b^
Walker 2017 [38], USA	Randomized controlled trial	Age: 12–18 years (*x̄* = 14.2, SD1.3), female 42%, serious mental health problems	**Wraparound**: team working with adolescents, their family members and the family’s social support network, determining the primary needs, service and support strategies to be included in the care plan **AMP**: Achieve My Plan, enhances Wraparound through multi-system involvement with caregivers and service providers Outpatient CAMHS	I: Wraparound with AMP: *n* = 35 C: Wraparound without AMP: *n* = 20	Primary outcomes: **Youth Participation in Planning Scale** (YPP): Youth participation in preparation and planning in favour of the intervention at 3–4 weeks and 10–12 weeks (*p* < 0.01). Accountability in favour of the intervention at 3–4 weeks (*p* < 0.03), but not at 10–12 weeks (*p* = 0.10) **Youth Empowerment Scale** (YES): No significant effects Secondary outcome: Intervention group participants were 2.35 times more likely to rate care planning meetings as much better than control group participants (*p* < 0.001)	**Internal validity**: Overall risk of bias: high Performance bias: high Selection, detection, reporting and other forms of bias: unclear **External validity**: More pragmatic than explanatory
Jager 2017 [34], ^**c**^ Netherlands	Longitudinal prospective cohort study	Age: 12–18 years (*x̄* = 15.2, SD1.7) female 61%, adolescents who signed up for psychosocial care (77% in mental health care)	**Psychosocial care**, mostly delivered by a mental healthcare organization (77%). Care with patient-centred communication, including shared decision-making. Intervention duration: 6 months Specialist healthcare services	T1 (baseline): *N* = 416 T2 (3mo.) + T3 (1 year): *n* = 315(76%)	**Strengths and Difficulties Questionnaire** (SDQ) with changes in Total Difficulties Score (TDS) from T1 to T3: Experience of shared decision-making associated with larger improvement in TDS scores, irrespective of adolescents’ expectations. Unmet shared decision-making communication needs associated with lower improvement in self-confidence (*p* < 0.001)	**Internal validity**: Overall risk of bias: high Selection, performance, detection and attrition bias: high. Reporting and other forms of bias: low **External validity**: Equally pragmatic and explanatory
Jager 2014 [33], Netherlands	Longitudinal prospective cohort study	Age: 12–18 years, female 65%, adolescents who signed up for psychosocial care (76% in mental health care)	**Psychosocial care**, mostly delivered by a mental healthcare organization (76%). Care with patient-centred communication, including shared decision-making Duration 3 months Specialist healthcare services	T1 (baseline): *N* = 416 T2 (3mo.): *n* = 211 (51%) (min. 2 appointments)	Shared decision-making on the **Consumer Quality Index** (CQI) at 3 months: Adolescents who considered shared decision-making to be important (expectations), but experienced it to less extent, had lower degree of improved understanding of mental health problems and how to handle them, compared to those who had agreement between expectations and experiences (OR 4.2, 95% CI 1.7–10.8, *p* < 0.01)	**Internal validity**: Overall risk of bias: high Selection, performance, detection and attrition bias: high. Reporting and other forms of bias: low **External validity**: Equally pragmatic and explanatory
Simmons 2017 [36], Australia	Non-randomized comparative study	Age: 16–25 years (*x̄* = 17.8, SD 2.9), female 63%, adolescents who attended a youth mental health service clinic	**Peer workers** engaged with adolescents during intake assessment and online shared decision-making tool, prior to individual counseling session with a clinician Historical comparison group without peer workers and online shared decision-making tool E-health in primary & secondary care	I: *n* = 149 Response to SDMQ-9: *n* = 78 (52%) C: *n* = 80 Response to SDMQ-9: *n* = 61(76%)	**Shared Decision-Making Questionnaire** (SDMQ-9) (clinician rated) on day 1: In favour of the intervention group (*p* = 0.015), but limited clinical effect (mean difference 2.4 on a 54 point scale)	**Internal validity**: Overall risk of bias: high Selection, performance, detection and attrition bias: high. Reporting and other forms of bias: unclear **External validity**: More pragmatic than explanatory
Simmons 2017 [37], Australia	Cohort study with pre- to post-assessment	Age: 12–25 years (*x̄* = 18.5, SD3.4), female 82%, depression (PHQ-9): mild (min.5 points)(18%), mild–moderate (26%), moderate–severe (56%)	**Online decision aid** to help adolescents make decisions in line with evidence and their personal preferences and values Primary care	T1 (before decision aid): *N* = 66 T2 (after decision aid): *n* = 57 (86%) T3 (8 weeks): *n* = 48 (73%)	**Patient Health Questionnaire** (PHQ-9) from T1 to T3: mean reduction of 2.7 points (95% CI, 1.3;4.0) **Decisional Conflict Scale** (DCS) from T1 to T2: mean reduction 17.8 points (95% CI 13.3;22.9, p < 0.001)	**Internal validity**: Overall risk of bias: high Selection, performance, detection, attrition and reporting bias: high. Other forms of bias: low **External validity**: More pragmatic than explanatory
Walker 2010 [39], USA	Cross-sectional and repeated measures survey	Age 14–21 years (*x̄* = 16.2,SD1.7), female 43%, mental health difficulties: ADHD, depression, bipolar disorder, PTSD, ODD, conduct disorder	**Testing of a Youth Empowerment Scale–Mental Health** (YES–MH), adapted from the Family Empowerment Scale (FES), services provided by multiple child- and family-serving agencies, primary & secondary care	T1 (baseline): *N* = 185 T2 (6 weeks): *n* = 60	Results based on exploratory factor analysis of YES–MH suggest three levels of empowerment: a) system: confidence & capacity to help providers improve services and help other youth with emotional/mental health difficulties b) services: confidence & capacity to work with service providers to select and optimize services c) self: confidence & capacity to cope with or manage one’s own condition Positive correlation between YES-MH and a 6-item Participation in Planning Scale (PPS)(*p* < 0.01)	**Internal reliability**: very good for both YES-MH (Cronbach’s alpha 0.85 – 0.91) and PPS (0.90) **Test–retest reliability** good for all three sub-scales of YES-MH (*p* < 0.01). No other forms of psychometric tests were applied
Nolkemper 2019 [35], Germany	Cross-sectional survey	Age: 12–18 years (*x̄* = 14.8, SD1.5), female 42%, adolescents who have been hospitalized for mental health conditions	**Psychiatric treatment** Child and adolescent psychiatry medical college & child and adolescent psychiatry university hospital	Experience of participation in psychiatric treatment: *N* = 114	Self-developed questionnaire focusing on feeling of being able to participate in decision-making (6 items, Likert scale): Yes, very much: 12% Yes: 40% Partially: 25% Not really: 13% Not at all: 10% No significant age, gender or clinic differences	**Internal validity**: Overall risk of bias: high Selection, performance, detection, attrition and other forms of bias: high. Reporting bias: low **External validity**: Equally pragmatic and explanatory

^a^I = Intervention, C = Control

^b^Internal validity: Cochrane Collaboration’s guidelines for risk of bias assessment [23]. External validity: The PRECIS tool for assessing studies on a pragmatic-explanatory continuum was used [26]. Validity assessment for Walker 2010 [39] focuses solely on criteria of relevance to psychometric tests

^c^Jager 2017 [34] builds on the same data as Jager 2014 [33], but assesses different outcomes and includes long-term follow-up

### Quality assessment of quantitative studies

The six studies reporting on user involvement at the individual level were all considered to have a high risk of bias, according to the Cochrane Collaboration’s guidelines [23]. The study assessing user involvement at the organizational level included two types of psychometric tests as part of assessing an outcome measure [39]. Three of the studies were, according to assessments carried out using the PRECIS tool [26], considered to be more pragmatic than explanatory [36–38]; and the remaining three were equally pragmatic and explanatory [33–35]. Further details are presented in Table 6.

### Experiences with user involvement

Thematic syntheses were carried out separately for user involvement at the individual and at the organizational level. Each theme is described and references to the research literature are provided.

#### User involvement at the individual level

The thematic synthesis of qualitative studies reporting on user involvement at the individual level resulted in three themes reported below: unilateral clinician control versus collaborative relationship; capacity and support for active involvement; and the right to be involved.

### Unilateral clinician control versus collaborative relationship

Adolescents’ involvement could be described as a dichotomy between unilateral control and collaborative relationship. Although a continuum of involvement could be envisaged, most study participants’ descriptions suggested that clinical practice involved either health personnel being in control of adolescents’ treatment and clinical decision-making, or adolescents becoming extensively involved in their own treatment and shared decision-making processes.

Health personnel’s unilateral control was found in several studies where adolescents described lack of control with limited possibilities to voice their opinion, limited treatment choices and limited involvement in decision-making processes [40–47]. Adolescents reported that health personnel exerted pressure and made decisions. This was illustrated by adolescents reporting that they did not feel heard [40, 42], feeling left out from meetings, being interrupted, ignored or not asked for their opinions; and pressured or forced to comply with health personnel’s decisions to attend meeting sessions, engage in uninteresting activities and to take medication [40, 43–47]. A perception of unilateral control could also result from receiving too little information about their health and treatment [42, 45]. Adolescents were reluctant to voice their opinions as they were only encouraged to express their views when they were consistent with health personnel’s perspectives and expressed in what was perceived to be an acceptable manner and at an appropriate time [40, 42]. Unilateral control could result when adolescents felt activities were not individually adapted, which prevented their participation [45]. Some health personnel were sceptical of the idea of handing over control to adolescents, whereas others were opposed to controlling and enforcing compliance of their young patients [43].

Other health personnel and adolescents described a collaborative therapeutic relationship throughout the entire treatment process [43, 48]. Collaborative relationships provided a framework that facilitated adolescents’ involvement. Key features of the framework included a good adolescent–practitioner relationship, open communication, and shared decision-making processes. As part of a collaborative relationship, health personnel offered adolescents context-appropriate choices, and regularly checked to what extent they wanted to be involved in decision-making processes. A good practitioner–adolescent relationship was essential as part of building trust [43–45, 49] and was characterized by open communication where health personnel shared their knowledge as professionals, as well as carefully listened to adolescents’ own experiences [42, 49, 50]. Adolescents could share their experiences with the use of medication and other aspects, which could help provide treatment options that were suitable for the individual adolescent [50]. Other examples of active collaboration included adolescents choosing their case manager, opting in or out of group participation, and engaging in various forms of treatment. The collaborative relationship, which included adolescents being offered choices in matters that affected their health care [43], contributed to shared decision-making processes [42–45, 49–52].

A collaborative relationship contributed to adolescents becoming more actively engaged in their treatment. The opposite, being left out of meetings, interrupted, ignored or not asked for input [45], or that health personnel exerted pressure and made unilateral decisions, contributed to distress and reducing their willingness to be involved in their treatment [40, 42–45, 47]. Not being involved in implementation of plans contributed to passive compliance and disengagement from the therapeutic process [53]. In a collaborative relationship where trust was established and adolescents received sufficient information, adolescents’ active participation in their therapy was facilitated [42–45, 49, 50, 54]. Adolescents’ involvement was associated with higher treatment attendance rates [54] and continuation of treatment; as opposed to treatment drop-out [44, 54]. Receiving enough information and support was associated with identifying treatment goals, self-care activities, and areas of decisional conflict [50]; practising ways to share information with health personnel [50]; and making informed choices for their healthcare [42].

### Capacity and support for active involvement

Across all study participants, capacity and support were experienced as key to adolescents’ involvement with different experiences and nuances conveyed among them. Capacity was described through adolescents’ ability to be involved and share their personal experiences, whereas health personnel’s capacity was described through available time and professional knowledge. Furthermore, practical or social support could strengthen adolescents’ involvement [45, 49, 50, 54–56].

The different studies provided conflicting evidence between and within adolescents themselves, caregivers, and healthcare staff on adolescents’ capacity to be actively involved in their healthcare [42, 43, 49, 53, 57]. Some health personnel considered adolescents’ young age, immaturity, symptoms or diagnoses, and lack of interest, to be potential barriers to involving them in their treatment and care in general, and in decision-making processes in particular [42, 43]. Moreover, other health personnel thought it was challenging to judge adolescents’ level of understanding due to medication effects [43]. Some suggested adolescents were not interested in attending and contributing to meetings [43], or were too depressed or lethargic to be actively involved in their treatment [49]. Contrary to these views, other health personnel [43] and adolescents themselves [53, 57] said they were interested in and motivated to be involved in decisions affecting their treatment. They wanted to be heard, and they had clear ideas about their care and the capacity to make sound judgements about it.

Health personnel’s capacity was questioned by adolescents. They reported that staff members were overwhelmed by their workload, thereby serving as a barrier to involvement of adolescents in their care [45, 57]. Adolescents’ involvement in treatment decision-making depended on the information they were provided by health personnel, which in turn was dependent on their professional competence about for example medication options, expected outcomes, side-effects and possible treatment choices [50].

Being informed about their health and treatment options was a typical form of practical support for adolescents’ active involvement [45, 49, 50, 54]. Too little information resulted in adolescents feeling lack of support through insufficient control and lack of motivation to be involved in their treatment [42, 45]. In order for information to be of help, it had to be relevant to adolescents [43]. Practical support could also involve enabling adolescents to come to consultations and limited transport options could reduce their possibilities to be more actively involved in appointments and activities [57]. However, adolescents also needed social support, for example by being heard, offered context-appropriate choices, and encouraged to actively participate in decision-making processes [43, 45, 49, 50, 54].

Although several studies suggested that adolescents wanted to be actively involved in decision-making processes, adolescents also expressed awareness of the challenges associated with being in a transitional phase, moving from childhood to adult life [42]. They sought support from others as part of the process, in particular by seeking information and guidance from parents and health personnel to help them make decisions [42, 49, 50, 52, 54]. According to adolescents, their parents and health personnel could support adolescents by sharing their professional knowledge and to provide them with tools such as shared decision worksheets [50], to help them make choices for their healthcare [52]. Adolescents were of the opinion that through a thorough exploration of their experiences, relationships, support networks, and views, health personnel would be in a better position to provide treatment options that were acceptable to adolescents and compatible with their cultural background [51, 52]. Health personnel could for example combine this with their professional knowledge to suggest treatment options other than medication [51].

### The right to be involved

A prerequisite for adolescents’ involvement in their treatment was a basic understanding of their inherent right to be involved [41–43, 49, 50, 53, 57]. Regardless of their age, adolescents wanted to be heard, their autonomy to be respected, and to be involved in decisions affecting their treatment, health, and wellbeing [41, 43, 48, 49, 53, 54, 57]. Although the degree of need for involvement varied between adolescents, they actively sought opportunities to be involved in decision-making processes [53], and expressed a wish to maintain some control through involvement in the patient–practitioner relationship [42]. They considered this to be essential to maintain their sense of autonomy [42, 49, 54]. Involvement could include for example decisions about their treatment plans [41, 43]; choice and change of therapist [43, 48, 54]; the time, length and frequency of treatment sessions [48, 54] and text message feedback solutions to express such wishes [58]; which family members who could attend meetings [48]; and the right to refuse health personnel’s proposals [54], including the use of medication [48]. Health personnel mostly shared adolescents’ views of their fundamental right to express their opinion, and they considered it a helpful contribution to treatment, although some healthcare personnel were sceptical about giving adolescents control of decisions related to their treatment [42, 43].

#### User involvement at the organizational level

The thematic synthesis of qualitative studies reporting on user involvement at the organizational level resulted in two themes reported below: involvement outcomes relevant to adolescents’ needs; and conditions for optimal involvement.

### Involvement outcomes relevant to adolescents’ needs

Involving adolescents at the organizational level should contribute to outcomes relevant to adolescents’ needs, seen from the perspectives of adolescents, caregivers, and health personnel. In general, participants expressed that adolescents’ involvement contributed to developing and improving mental health services or that it had the potential to do so [40, 56, 59–63]. More specifically, this included the development and use of terminology and models for mental health and participation relevant to adolescents [56, 59, 62]. The design and contents of interventions and psychoeducational resources should reflect adolescents’ experiences and needs [63]. Allowing adolescents the opportunity to influence the design and implementation of treatment programmes and interventions to improve treatment outcomes helped strengthen the relevance, appropriateness, and acceptability of the treatment [60–63]. Adolescents’ involvement did also contribute to change the treatment environment so that it was better adapted to adolescents’ needs, for example to make conference rooms less formal [40]. Adolescents’ perspectives did also contribute to improve the content of health personnel’s training, and the relevance and quality of the services [60].

Adolescent consultants contributed to empower other adolescents with mental health challenges to take charge of their recovery through, e.g. educational or one-on-one support. They helped other adolescents and parents to identify their goals, self-care activities, and areas of decisional conflict [50], as well as to negotiate the patient–practitioner relationship when adolescents did not get along with their counsellor [55]. Implications for adolescents who participated as consultants could potentially also support their sense of autonomy and self-efficacy and empower them to take charge of their own recovery. Moreover, they could gain work experience, build professional and social skills, and expand their networks within the context of a safe environment with proper support [55, 59].

### Conditions for optimal involvement

Conditions for adolescents’ optimal involvement at the organizational level reported across study participants included openness to adolescents’ viewpoints and understandings, clarity of roles, information provision, autonomy, skills training, backgrounds and personal experiences, diversity, and formal recognition of efforts to involve adolescents [45, 50, 53, 55, 56, 59, 61, 63].

In order to involve adolescents at the organizational level, professionals needed to be open to adolescents’ viewpoints and understandings of mental health [56], and clear definition and clarity of roles should be agreed and described to understand the boundaries and limitations of adolescents’ involvement [50, 59, 61].

Adolescents also thought involvement could be optimized by information provided for them about existing services and projects which they could be involved in [45, 50]. By being given the freedom to identify and make decisions about projects they cared about and could run themselves [45, 50, 56, 61], adolescents could make autonomous decisions about whether and the extent to which they wanted to be involved [50].

Skills training supported optimal involvement of adolescents at the organizational level. Such training could introduce roles, tools, and methods for shared decision-making processes [50, 59]. Tools could for example include methods to formalize and facilitate shared decision-making while still allowing flexibility for the individual needs of adolescents and parents [50].

Adolescents having personal experiences with the mental health services could optimize their involvement at the organizational level, particularly if they worked directly with other adolescents as peer consultants, so that they could better understand their concerns and needs [59, 61]. Adolescents who had received hospital treatment for serious mental illnesses had clear ideas concerning rules, regulations, and treatment and could make sound judgments about such treatment [53]. Furthermore, adolescents with diverse backgrounds and those from “disadvantaged” backgrounds would strengthen the diversity in perspectives and increase the likelihood that changes to services would be relevant to adolescents of, e.g. ethnic minority backgrounds [55, 59].

Formal recognition of adolescents’ contribution as consultants was to provide payment as employees rather than involving them as volunteers, and to enable them to work both independently and be involved in group activities [59]. Adolescents pointed out that leaders could recognize staff who encouraged user involvement, organized workshops, and discussed their experiences, as well as communicate the benefits of user involvement within their clinics [50].

### Effectiveness of user involvement

A narrative report of results of individual studies on the effectiveness of user involvement is presented in text and in tabular form (Table 6). The articles reporting on individual studies are presented according to the level of evidence associated with their research design (from randomized controlled trials to cross-sectional surveys). No synthesis of data is presented due to heterogeneity of interventions and outcomes.

#### User involvement at the individual level

Out of six identified articles, three assessed the effectiveness of additional support to facilitate adolescents’ involvement in their own care [36–38]; one assessed the effectiveness of shared decision-making on adolescents’ ability to handle mental health problems in the short term [33] and their overall strengths, difficulties and self-confidence in the longer term [34]; and one reported the prevalence of adolescents’ participation in decision-making [35].

The results of a randomized controlled trial suggest that additional support provided by a team working with adolescents with severe mental health problems, their family and social support network in developing a care plan, may increase youth participation in treatment planning, both in the short (3–4 weeks) and longer term (10–12 weeks) (*p* < 0.01) [38]. These adolescents were more than twice as likely to positively rate care planning meetings, compared to those in the control group (*p* < 0.001).

A non-randomized controlled study that took place in a youth mental health service clinic found a significant effect, measured using the Shared Decision-Making Questionnaire (SDMQ-9) (*p* = 0.015), of a combination of peer workers engaged with adolescents at intake assessment together with an online shared decision-making tool prior to counselling sessions, compared to a historical comparison group [36]. The results did, however, only suggest a small clinical effect.

Results of a cohort study suggested that adolescents who had higher expectations but poorer experiences of shared decisions-making in psychosocial care had lower degrees of understanding of and ability to handle mental health problems at 3 months, compared to adolescents whose experiences corresponded with their expectations (OR 4.2, 95% CI 1.7–10.8, *p* < 0.01) [33]. In the long-term follow-up at one year, shared decision-making was associated with significant changes in adolescents’ Total Difficulties Score (TDS), measured using the Strengths and Difficulties Questionnaire (SDQ) [34]. Although results were then irrespective of adolescents’ expectations, improvement in self-confidence was lower when communication needs were not met (*p* < 0.001).

An online aid aimed at supporting adolescents with mild to severe depression in making decisions in line with their values and preferences, as well as in line with the existing research evidence, was tested in a cohort study from baseline to eight weeks [37]. At 8 weeks, results showed a statistically significant reduction in depression (PHQ-9) scores, although the clinical importance of this was uncertain (mean change 2.7 points, 95% CI, 1.3;4.0). Significant improvements were also found on the Decisional Conflict Scale (DCS) from before to after use of the decision aid (mean change 17.8 points, 95% CI 13.3;22.9).

Results of a cross-sectional survey suggested that over half of adolescents who had been hospitalized for mental health conditions felt they were able to participate in decision-making processes, whereas one quarter felt they could participate partially and one quarter not at all [35].

#### User involvement at the organizational level

A cross-sectional and repeated measures survey reported on results of testing a Youth Empowerment Scale–Mental Health (YES-MH) for adolescents with various mental health difficulties [39]. The survey measured adolescents’ participation in team-based services and treatment planning for mental health services. Results of a factor analysis suggested empowerment of adolescents through their confidence and capacity to work with service providers to select and optimize services; to help providers improve services; and to help other youth with mental health difficulties.

### Safety associated with user involvement

No study aimed to report on the safety associated with user involvement in adolescents’ mental healthcare. A few studies did however report on issues that potentially could influence the safety of adolescents’ mental healthcare. Examples included a qualitative study where involvement of adolescents in decision-making was thought to be a potential threat to their safety by some health personnel [43]. Their arguments did, however, not pertain to the individual youths’ capability or competence to participate in decision-making, but could be understood as exercising undue professional power as they considered adolescents not to be competent in making decisions for their mental healthcare irrespective of their arguments, state of health or level of maturity. In a second qualitative study, some staff expressed concerns about risks associated with involvement of adolescent consultants. For example, the experience of being a consultant was thought by some staff to potentially be overwhelming to adolescents and could serve as a barrier to their own recovery [59]. Healthcare personnel thought these adolescents could also misunderstand conversations between staff and thereby breach confidentiality.

## Discussion

This systematic review of user involvement in adolescents’ mental healthcare demonstrates that the current literature is dispersed and fragmented. There is weak evidence for the effectiveness of user involvement using quantitative research designs, while there is more evidence for the experiences with user involvement using qualitative research methods. There is hardly any evidence addressing safety issues associated with user involvement. User involvement at the individual level is more often reported in studies than user involvement at the organizational level.

The results of the review leave little doubt that adolescents want to be involved in decisions affecting their mental healthcare, thereby confirming earlier findings [e.g. 14]. However, user involvement of adolescents in mental healthcare at the individual level takes many forms and with different experiences ranging from “just” being heard about their opinion to being involved in decision-making processes. In a recent systematic review, factors influencing person-centred care were examined, recommending greater focus on the role of relationships, service information, and support and training for professionals [64]. These factors are supported by the current review results, and in particular by the clear evidence of positive experiences related to collaborative relationships.

The effectiveness of adolescents’ involvement at the individual level is still not established in the research literature besides preliminary findings in a few interventional studies showing evidence for certain tools related to support care planning meetings, intake assessment, and shared decision-making [36–38]. Some evidence was also found suggesting that involvement in decision-making could potentially contribute to improved mental health outcomes [33, 34]. These results should, however, be interpreted with caution, both due to the limited number of identified studies and due to high risk of bias. Results of another systematic review suggested that interventions considering barriers at several levels (individual, family, community, organization) were effective in supporting adolescents’ engagement in mental healthcare [65]. However, this review examined attendance, rather than user involvement.

User involvement of adolescents in mental healthcare at the organizational level is less commonly reported in the literature but shares similarities with the individual level concerning the conditions of information, openness, and support structures. Distinct organizational level issues were related to the need for skills training, clarity of roles and the inclusion of adolescents of different backgrounds and with different experiences. The preliminary evidence suggests such involvement at the organizational level could potentially contribute to development of outcomes of relevance to adolescents’ needs [39].

The paucity of research evidence assessing safety issues associated with user involvement in adolescents’ mental healthcare is striking. However, the World Health Organization suggests that involvement of patients may be fundamental to improve patient safety [12]. Our review found a lack of literature exploring safety issues of how adolescents may be involved to improve patient safety. The sparse literature suggests that professionals doubt adolescents’ capacity to be involved due to age and severity of symptoms, in particular during a mental health crisis [64]. We identified a single study [43] indicating that organizational culture and paternalistic approaches may affect professionals’ perception of adolescents’ involvement as a safety issue. It is unclear why there is such a paucity of safety research focusing on user involvement in adolescents’ mental healthcare given the importance of patient safety for example for adolescents who self-harm, who have suicidal thoughts or plans, or eating disorders. Practitioners’ concerns about potential adverse events and deterioration of adolescents’ mental health might explain their reluctance to involve them in decision-making processes. This could, e.g. include adolescents right to refuse medication. However, an opposing argument may be that more active involvement of adolescents in their mental healthcare could strengthen their trust in practitioners and therapies offered and to increase treatment compliance. This would also be more in line with a recovery-oriented approach supporting adolescents’ active engagement and health-promoting involvement. Where involvement in decision-making could pose a potential threat to adolescents’ safety, alternative approaches to involving adolescents could be suggested, e.g. to inform about and discuss the reasons for not giving them decision-making power. More research is needed to test hypotheses and to map safety issues and barriers to user involvement both at the individual and organizational level. Additional research is further needed to determine how to tailor user involvement so that adolescents with variable capacities can safely participate in their treatment.

### Strengths and limitations

It cannot be ruled out that the applied review procedures contributed to overseeing relevant studies, as there seems to be no standardized search terminology associated with the field of adolescents’ involvement. This is suggested as half of the identified literature was found through a single source and no single database identified more than half of the studies. The use of search terminology in other languages might have helped to identify more articles published in the non-English literature. However, the review considerably expands past limited research-based knowledge about involvement in adolescents’ mental healthcare [e.g. 15], and we consider it a strength to have used a substantial number of databases, a broad range of search terms, and that two researchers carried out all parts of the search processes.

The age limitation (13–18 years) may have resulted in exclusion of studies focusing on young adults that could be of relevance to teenagers. However, the applied age range was selected in order to specifically focus on an under-investigated group of adolescents who are in a phase in life where they may have varying degrees of decision-making rights depending on national legislation and regulation.

The heterogeneity of identified studies resulting from different research designs and different outcomes is not surprising, due to the use of wide inclusion criteria. However, the limited amount of identified literature precluded development of recommendations for any sub-groups of adolescents, for example according to ethnicity or diagnostic groups. Moreover, no conclusions can be drawn about safety issues associated with user involvement, as hardly any research addressed such issues. Nevertheless, a strength of the systematic review is that the results of the meta-synthesis provide insight into the experiences of user involvement in adolescents’ mental healthcare. Furthermore, adolescent co-researchers were involved in the analytic process in order to also include their perspectives.

### Recommendations

Mental healthcare services should facilitate user involvement to promote treatment attendance and adherence. Guidelines for strengthening the collaborative practitioner–adolescent relationship should be developed with input from adolescents, including those with ethnic and other minority backgrounds, as well as healthcare practitioners. Guidelines should include suggestions for questions for reflection as part of the patient–practitioner relationship to strengthen adolescents’ active involvement in their own healthcare. Issues to be included could be adolescents’ values and preferences; the extent and ways in which they want to be involved in their own treatment; the persons who may represent them when they do not want to be actively involved; adolescents’ treatment preferences in the event they are only to a limited extent able to participate in decision-making (e.g. in psychotic phases); the means and purposes of their involvement in decision-making processes. Facilitators to user involvement beyond capacity and support should be further explored. We suggest involving adolescents with diverse backgrounds at an organizational level when developing and improving services to strengthen the relevance of mental health services.

Although involvement of adolescents in their mental healthcare should be considered a human right and also a legal obligation, there is considerable need for more research-based knowledge about the best ways in which adolescents want to and can be involved in their mental healthcare. Research should assess the effectiveness of user involvement in adolescents’ mental healthcare, using outcomes of clinical relevance and of relevance to adolescents themselves. Safety research should particularly address issues of involvement for at-risk groups, to identify the extent and ways in which these adolescents can best be involved in decisions affecting their mental healthcare. Further research is also needed to explore how user involvement can best be adapted to different sub-groups, for example for various ethnic and minority groups. Moreover, further knowledge is needed on how to strengthen the facilitators and limit the barriers to user involvement, including support and training for healthcare personnel.

## Conclusion

By systematically reviewing the literature, we have established the current knowledge evidence on the experiences with, the effectiveness of, and the safety associated with user involvement in adolescents’ mental healthcare. Results identify a variety of experiences at the individual level related to the continuum between unilateral control exerted by clinicians and a collaborative practitioner–adolescent relationship; the key of capacity and support for adolescents’ involvement; and the prerequisite of a basic understanding of adolescents’ inherent right to be involved. Less experiences are identified related to the organizational level, yet the need to ensure relevant outcomes of involvement for adolescents was established as a vital parameter, as well as a set of conditions for optimal involvement. The effectiveness of user involvement is less clear in the current literature, yet some preliminary evidence is established related to interventions such as support for care planning meetings, intake assessment, and shared decision-making. Evidence for potential safety issues associated with adolescents’ user involvement is currently not established in the research literature. The results of the reported literature review warrant future research within the areas of organizational level involvement of adolescents, the development and effectiveness of different measures for adolescents’ user involvement, and how involvement of adolescents may influence patient safety.

## Supplementary Information

Below is the link to the electronic supplementary material.

Supplementary file 1 (DOCX 27 KB)

Supplementary file 2 (DOCX 14 KB)

## Data Availability

The research materials can be accessed by contacting the corresponding authors.
