# Supplementary material for: User involvement in adolescents’ mental healthcare: a systematic review
Source: Eur Child Adolesc Psychiatry. 2021 Jun 5;31(11):1765–88. doi: 10.1007/s00787-021-01818-2 (PMC9666298; doi:10.1007/s00787-021-01818-2)
Supplement: Supplementary file 1 — (DOCX 27 KB) [file 787_2021_1818_MOESM1_ESM.docx]

**Full electronic search strategy – EMBASE**

First search: 31.07.2017. Update search: 18.06.2019

**First search**: 31.07.2017

1. adolescent psychology.mp. or exp child psychology/ 16,118

2. adolescent psychiatry.mp. or exp child psychiatry/ 20,736

3. 1 OR 2 36,285

4. exp personal autonomy/

5. exp patient autonomy/ 16,367

6. exp consultation/ 90,476

7. exp decision making/ 300,674

8. exp empowerment/ 5,733

9. exp patient participation/ 166,837

10. 4 OR 5 OR 6 OR 7 OR 8 OR 9 418,127

11. 3 AND 10 1,962

12. limit 11 to adolescent <13 to 17 years> **873**

13 "adolescen*".m_titl. 162,107

14. "teen*".m_titl. 11,762

15. "youth*".m_titl. 25,447

16. 13 OR 14 OR 15 197,376

17. mental.m_titl. 94,876

18. "psycholog*".m_titl. 61,525

19. "psychiatr*".m_titl. 97,700

20. 17 OR 18 OR 19 248,043

21. "autonom*".m_titl. 33,718

22. client-centred.m_titl. 169

23. "collaborat*".m_titl. 28,373

24. "consultat*".m_titl. 16,207

25. "contribut*".m_titl. 117,283

26. decision making.m_titl. 24,304

27. "empower*".m_titl. 4,794

28. "engag*".m_titl. 14,010

29. governance.m_titl. 3,756

30. "inclusi*".m_titl. 16,149

31. information sharing.m_titl. 245

32. "involv*".m_titl. 179,052

33. mutual agreement.m_titl. 4

34. "negotiat*".m_titl. 2,955

35. "opinion*".m_titl. 15,745

36. patient-centred.m_titl. 977

37. "participat*".m_titl. 34,911

38. "partnership*".m_titl. 9,866

39. perspective*.m_titl. 117,135

40. peer support.m_titl. 875

41. self-determination.m_titl. 945

42. 21 OR 22 OR 23 OR 24 OR 25 OR 26 OR 27 OR 28 OR 29 OR 30 OR 31 OR 32 OR 33 OR 34 OR 35 OR 36 OR 37 OR 38 OR 39 OR 40 OR 41 54 612,191

43. 16 AND 20 AND 42 **515**

44. limit 11 to adolescent <13 to 17 years> **361**

45. 12 OR 44 **1,205**

**Result of EMBASE search 31.07.2017: 1,205**

-----------------------------------------------------------------------------------

**EMBASE update search**

**Update search**: 18.06.2019

1. adolescent psychology.mp. or exp child psychology/ 15,285

2. adolescent psychiatry.mp. or exp child psychiatry/ 21,970

3. 1 OR 2 36,740

4. exp personal autonomy/ 13,015

5. exp patient autonomy/ 5,286

6. exp consultation/ 107,821

7. exp decision making/ 345,322

8. exp empowerment/ 7,819

9. exp patient participation/ 25,368

10. 4 OR 5 OR 6 OR 7 OR 8 OR 9 483,589

11. 3 AND 10 2,156

12. limit 11 to adolescent <13 to 17 years> **955**

13 "adolescen*".m_titl. 187,332

14. "teen*".m_titl. 13,094

15. "youth*".m_titl. 39,988

16. 13 OR 14 OR 15 229,142

17. mental.m_titl. 105,075

18. "psycholog*".m_titl. 67,456

19. "psychiatr*".m_titl. 103,052

20. 17 OR 18 OR 19 268,921

21. "autonom*".m_titl. 36,519

22. client-centred.m_titl. 178

23. "collaborat*".m_titl. 33,440

24. "consultat*".m_titl. 16,257

25. "contribut*".m_titl. 122,275

26. decision making.m_titl. 29,261

27. "empower*".m_titl. 5,905

28. "engag*".m_titl. 18,563

29. governance.m_titl. 4,348

30. "inclusi*".m_titl. 17,532

31. information sharing.m_titl. 299

32. "involv*".m_titl. 196,147

33. mutual agreement.m_titl. 5

34. "negotiat*".m_titl. 3,213

35. "opinion*".m_titl. 17,008

36. patient-centred.m_titl. 1,273

37. "participat*".m_titl. 39,879

38. "partnership*".m_titl. 11,216

39. perspective*.m_titl. 135,295

40. peer support.m_titl. 1,177

41. self-determination.m_titl. 1,080

42. 21 OR 22 OR 23 OR 24 OR 25 OR 26 OR 27 OR 28 OR 29 OR 30 OR 31 OR 32 OR 33 OR 34 OR 35 OR 36 OR 37 OR 38 OR 39 OR 40 OR 41 54 681,527

43. 16 AND 20 AND 42 **662**

44. limit 11 to adolescent <13 to 17 years> **472**

45. 12 OR 44 **1,395**

46. Publication Year 2017 to 2019 **223**

**Result of EMBASE search 18.06.2019: 223**
